# Supplementary material for: Polylogarithmic-depth controlled-NOT gates without ancilla qubits
Source: Nat Commun. 2024 Jul 13;15:5886. doi: 10.1038/s41467-024-50065-x (PMC11246449; doi:10.1038/s41467-024-50065-x)
Supplement: Supplementary file 1 — Supplementary Information [file 41467_2024_50065_MOESM1_ESM.pdf]

Supplementary Information for: Polylogarithmic-depth controlled-NOT gates without ancilla qubits

Baptiste Claudon,[1,2,*] Julien Zylberman,[3] César Feniou,[1,2] Fabrice Debbasch,[3] Alberto Peruzzo,[1] and Jean-Philip Piquemal[1,2,†]

[1] *Qubit Pharmaceuticals, Advanced Research Department, 75014 Paris, France*
[2] *Sorbonne Université, LCT, UMR 7616 CNRS, 75005 Paris, France*
[3] *Sorbonne Université, Observatoire de Paris, Université PSL, CNRS, LERMA, 75005 Paris, France*

SUPPLEMENTARY NOTE 1 - STEP BY STEP STUDY OF THE PROPOSED QUANTUM CIRCUITS

Further notations. Before proving the correctness of the method, further notations need to be introduced. The operator $\oplus : \{0,1\}^2 \to \{0,1\}$ is used to denote addition modulo 2, while $\boxplus : \cup_{n=1}^{\infty}\{0,1\}^n \times \{0,1\}^n \to \cup_{n=1}^{\infty}\{0,1\}^n$ signifies bitwise addition modulo two: $(x_1, ..., x_n) \boxplus (y_1, ..., y_n) = (x_1 \oplus y_1, ..., x_n \oplus y_n)$. Let $c : \cup_{n=1}^{\infty}\{0,1\}^n \to \{0,1\}, (x_1, ..., x_n) \mapsto \prod_{j=1}^{n} x_j$. Also, let $\bar{c} : \cup_{n=1}^{\infty}\{0,1\}^n \to \{0,1\}, (x_1, ..., x_n) \mapsto \prod_{j=1}^{n}(1 - x_j)$. It will be practical to denote with the same symbol R a set of qubits and the bistring associated with a computational basis state label in the $|\cdot\rangle$ notation. The operations c and $\bar{c}$ will turn out to be very useful since for any multi-qubit register R, target qubit register t and one of their computational basis states $|R, t\rangle$: $\mathcal{C}_R^t |R, t\rangle = |R, t \oplus c(R)\rangle$ and $\mathcal{C}_{\bar{R}}^t = |R, t \oplus \bar{c}(R)\rangle$. For an ancilla qubit a, it also holds that $\mathcal{C}_{\bar{R}\cup a}^t |R, a, t\rangle = |R, a, t \oplus c(a)\bar{c}(R)\rangle$.

Let $n > 4$ and $p = \lfloor\sqrt{n}\rfloor$. Again, write $R = \{q_j\}_{j=0}^{n-1}$. Let $R \supset R_0 = \{q_i\}_{i=0}^{2p-1}$ be a first subregister of $2p$ control qubits, and for each $i \geq 1$, let $R \supset R_i = \{q_j \text{ if } j \leq n\}_{j=(1+i)p}^{(2+i)p-1}$ be other subregisters of size at most p. Let $R_0^* = \{q_i \in R_0 : R_{i+1} \neq \emptyset\}$ and $R_0' = R_0 \backslash R_0^*$. Finally, let $b = |R_0^*|$.

The analysis of the construction follows the steps given below.

- First, the zeroed-ancilla circuit is proven to be correct.
- Second, the borrowed-ancilla circuit is proven to be correct.
- Third, it is shown that each of the smaller multi-controlled NOT appearing in the borrowed-ancilla circuit can be achieved with the help of a locally borrowed ancilla.
- Fourth, the recursion characterising the circuit depth is solved.

A. Analysis of $\mathcal{U}_0$

The single-zeroed-ancilla circuit corresponds to the unitary operation

$$\mathcal{U}_0 = \mathcal{C}_{R_0}^a \left(\prod_{i=1}^{b} \mathcal{C}_{R_i}^{q_{i-1}}\right) \mathcal{C}_{\overline{R_0^*}\cup a}^t \left(\prod_{i=1}^{b} \mathcal{C}_{R_i}^{q_{i-1}}\right) \mathcal{C}_{R_0}^a = \mathcal{C}_{R_0}^a \mathfrak{C}\, \mathcal{C}_{R_0}^a. \tag{1}$$

Proving that $\mathcal{U}_0$ performs the desired operation relies on the following lemma.

Lemma 1. It holds that:

$$c(R_0)\bar{c}(R_0^* \boxplus (c(R_1), ..., c(R_b))) = c(R). \tag{2}$$

It can be noted that $R_0^* \boxplus (c(R_1), ..., c(R_b)) = (q_0 \oplus c(R_1), ..., q_{b-1} \oplus R_b)$.

---

* baptiste.claudon@qubit-pharmaceuticals.com
† jean-philip.piquemal@sorbonne-universite.fr

Proof. Assume that $c(R) = 1$. Necessarily all the qubits in R are in state 1 and for each $i \in \{0, ..., b\}$, R_i verifies $c(R_i) = 1$. In particular, for each $i \in \{1, ..., b\}$, $q_{i-1} \oplus c(R_i) = 1 \oplus 1 = 0$. As a consequence, $\bar{c}(R_0^* \boxplus (c(R_1), ..., c(R_b))) = \bar{c}(q_0 \oplus R_1, q_1 \oplus R_2, ..., q_{b-1} \oplus R_b) = \prod_{i=1}^{b} 1 = 1$. Summarising:

$$c(R) = 1 \implies c(R_0)\bar{c}(R_0^* \boxplus (c(R_1), ..., c(R_b))) = 1 = c(R). \tag{3}$$

Now, assume that $c(R) = 0$. If $c(R_0) = 0$, then $c(R_0)\bar{c}(R_0^* \boxplus (c(R_1), ..., c(R_b))) = 0 = c(R)$ and we are done. Thus, assume that $c(R_0) = 1$. Since $c(R) = 0$, there must exists a qubit $q_k \in R \backslash R_0$ in state $|0\rangle$. Let $j \in \{1, ..., b\}$ be the register index such that $q_k \in R_j$. Necessarily, $c(R_j) = 0$. However, since $q_{j-1} \in R_0$, $q_{j-1} \oplus c(R_j) = q_{j-1} = 1$. It follows that:

$$\bar{c}(R_0^* \boxplus (c(R_1), ..., c(R_b)) = (1 - (q_{j-1} \oplus c(R_j))) \prod_{l=1, l\neq j}^{b} (1 - (q_{l-1} \oplus R_l)) = 0 \prod_{l=1, l\neq j}^{b} (1 - (q_{l-1} \oplus R_l)) = 0. \tag{4}$$

Summarising,

$$c(R) = 0 \implies c(R_0)\bar{c}(R_0^* \boxplus (c(R_1), ..., c(R_b)) = 0 = c(R). \tag{5}$$

Therefore, in any case, $c(R) = c(R_0)\bar{c}(R_0^* \boxplus (c(R_1), ..., c(R_b))$.

Let us also compute the action of $\mathfrak{C}$ on an arbitrary computational basis state $|R_0^*, R_0', R\backslash R_0, a, t\rangle$.

Lemma 2. The following equation holds for any computational basis state $|R_0^*, R_0', R\backslash R_0, a, t\rangle$.

$$\mathfrak{C} |R_0^*, R_0', R\backslash R_0, a, t\rangle = |R_0^*, R_0', R\backslash R_0, a, t \oplus a\bar{c}(R_0^* \boxplus (R_1, ..., R_b))\rangle . \tag{6}$$

Proof.

$$\begin{aligned}
\mathfrak{C} |R_0^*, R_0', R\backslash R_0, a, t\rangle &= \left(\prod_{i=1}^{b} \mathcal{C}_{R_i}^{q_{i-1}}\right) \mathcal{C}_{\overline{R_0^*} \cup a}^{t} \left(\prod_{i=1}^{b} \mathcal{C}_{R_i}^{q_{i-1}}\right) |R_0^*, R_0', R\backslash R_0, a, t\rangle \\
&= \left(\prod_{i=1}^{b} \mathcal{C}_{R_i}^{q_{i-1}}\right) \mathcal{C}_{\overline{R_0^*} \cup a}^{t} |R_0^* \boxplus (c(R_1), ..., c(R_b)), R_0', R\backslash R_0, a, t\rangle \\
&= \left(\prod_{i=1}^{b} \mathcal{C}_{R_i}^{q_{i-1}}\right) |R_0^* \boxplus (c(R_1), ..., c(R_b)), R_0', R\backslash R_0, a, t \oplus c(a)\bar{c}(R_0^* \boxplus (R_1, ..., R_b))\rangle \\
&= |R_0^*, R_0', R\backslash R_0, a, t \oplus c(a)\bar{c}(R_0^* \boxplus (R_1, ..., R_b))\rangle .
\end{aligned} \tag{7}$$

Conclude by noticing that for a single-qubit labelled by a in a state of the computational basis, $a = c(a)$.

Let us verify that for any computational basis state of the form $|R_0^*, R_0', R\backslash R_0, 0, t\rangle$, $\mathcal{U}_0 |R_0^*, R_0', R\backslash R_0, 0, t\rangle = \mathcal{C}_R^t |R_0^*, R_0', R\backslash R_0, 0, t\rangle$.

$$\begin{aligned}
\mathcal{U}_0 |R_0^*, R_0', R\backslash R_0, 0, t\rangle &= \mathcal{C}_{R_0}^a \mathfrak{C}\, \mathcal{C}_{R_0}^a |R_0^*, R_0', R\backslash R_0, 0, t\rangle \\
&= \mathcal{C}_{R_0}^a \mathfrak{C} |R_0^*, R_0', R\backslash R_0, c(R_0), t\rangle && \text{by definition of } C^n(X) \text{ and } c \\
&= \mathcal{C}_{R_0}^a |R_0^*, R_0', R\backslash R_0, c(R_0), t \oplus c(R_0)\bar{c}(R_0^* \boxplus (R_1, ..., R_b))\rangle && \text{by Lemma 2} \\
&= |R_0^*, R_0', R\backslash R_0, 0, t \oplus c(R_0)\bar{c}(R_0^* \boxplus (R_1, ..., R_b))\rangle && \text{by definition of } C^n(X) \text{ and } c \\
&= |R_0^*, R_0', R\backslash R_0, 0, t \oplus c(R)\rangle && \text{by Lemma 1} \\
&= \mathcal{C}_R^t |R_0^*, R_0', R\backslash R_0, 0, t\rangle .
\end{aligned} \tag{8}$$

By linearity, $\mathcal{U}_0$ performs the right operation on any superposition of the computational basis with ancilla in state $|0\rangle$, hence any state with zeroed ancilla.

B. Analysis of $\mathcal{U}$

Following the same logic, the correctness of single-borrowed-ancilla circuit is proven. This circuit acts as:

$$\mathcal{U} = \left(\prod_{i=1}^{b} \mathcal{C}_{R_i}^{q_{i-1}}\right) \mathcal{C}_{\overline{R_0^*}\cup a}^{t} \left(\prod_{i=1}^{b} \mathcal{C}_{R_i}^{q_{i-1}}\right) \mathcal{U}_0 = \mathfrak{C}\, \mathcal{U}_0. \tag{9}$$

The evolution of an arbitrary input state $|R_0^*, R_0', R\backslash R_0, a, t\rangle$ is given in equation 10.

$$\begin{aligned}
\mathcal{U}\,|R_0^*, R_0', R\backslash R_0, a, t\rangle &= \mathfrak{C}\, \mathcal{C}_{R_0}^{a} \mathfrak{C}\, \mathcal{C}_{R_0}^{a}\, |R_0^*, R_0', R\backslash R_0, a, t\rangle \\
&= \mathfrak{C}\, \mathcal{C}_{R_0}^{a} \mathfrak{C}\, |R_0^*, R_0', R\backslash R_0, a \oplus c(R_0), t\rangle \\
&= \mathfrak{C}\, \mathcal{C}_{R_0}^{a}\, |R_0^*, R_0', R\backslash R_0, a \oplus c(R_0), t \oplus (a \oplus c(R_0))\bar{c}(R_0^* \boxplus (c(R_1), ..., c(R_b)))\rangle && \text{by Lemma 2} \\
&= \mathfrak{C}\, \mathcal{C}_{R_0}^{a}\, |R_0^*, R_0', R\backslash R_0, a \oplus c(R_0), t \oplus c(R) \oplus a\bar{c}(R_0^* \boxplus (c(R_1), ..., c(R_b)))\rangle && \text{by Lemma 1} \\
&= \mathfrak{C}\, |R_0^*, R_0', R\backslash R_0, a, t \oplus c(R) \oplus a\bar{c}(R_0^* \boxplus (c(R_1), ..., c(R_b)))\rangle \\
&= |R_0^*, R_0', R\backslash R_0, a, t \oplus c(R) \oplus 2a\bar{c}(R_0^* \boxplus (c(R_1), ..., c(R_b)))\rangle && \text{by Lemma 2} \\
&= |R_0^*, R_0', R\backslash R_0, a, t \oplus c(R)\rangle \\
&= \mathcal{C}_{R}^{t}\, |R_0^*, R_0', R\backslash R_0, a, t\rangle\,.
\end{aligned} \tag{10}$$

By linearity, $\mathcal{U} = \mathcal{C}_R^t$.

C. Each smaller multi-controlled NOT gate has a borrowed ancilla at its disposal

Lemma 3. The register R_0' has more qubits than the register R_0^*: $|R_0'| \geq |R_0^*|$.

Proof. $|R\backslash R_0| = n - 2p < (p+1)^2 - 2p = p^2 + 1$. Thus, $|R\backslash R_0| \leq p^2$. In words, the qubits in $R\backslash R_0$ can be gathered in at most p subregisters of size p. In equation, $|R_0^*| \leq p$. Finally, $|R_0'| = |R_0| - |R_0^*| \geq 2p - p = p \geq |R_0^*|$.

As a consequence, each of the factors in

$$\prod_{i=1}^{b} \mathcal{C}_{R_i}^{q_{i-1}} \tag{11}$$

can be executed with the help of a borrowed ancilla in R_0'.

D. Solving the recursion

The operation with the highest number of control qubits is controlled by the state of R_0, of size $|R_0| = 2p$. It is applied twice. A layer of $C^p(X)$ gates is applied four times, and a layer of $C^{p+1}(X)$ (or even $C^p(X)$ in some cases) gates is applied twice. The overall depth can be uniformly bounded by the depth of eight successive $C^{2p}(X)$ gates plus a constant term corresponding to the layers of X gates required by the white controls. Let us conclude the analysis by upper bounding the resulting depth. Let $D(n)$ be the depth of circuit $C^n(X)$. Assume that $n = 2^{k+2}$,

$$D\left(2^{k+2}\right) \leq 2D\left(2^{\frac{k+2}{2}+1}\right) + 4D\left(2^{\frac{k+2}{2}}\right) + 2D\left(2^{\frac{k+2}{2}}+1\right) + 4 \leq 8D\left(2^{\frac{k+2}{2}+1}\right) + 4 = 8D\left(2^{\frac{k}{2}+2}\right) + 4 \in \mathcal{O}\left(k^{\log(8)}\right) \tag{12}$$

by the Master Theorem 1. In terms of n, the depth of the n-control gate is $\mathcal{O}\left(\log(n)^3\right)$.

SUPPLEMENTARY NOTE 2 - MASTER THEOREM

The Master Theorem 1 is a fundamental theorem for the analysis of dynamical programming algorithms [1]. The master method, based on this theorem, provides asymptotic growths for recurrences of the form $T(n) = aT(n/b) + f(n)$, where $a \geq 1$, $b > 1$.

Theorem 1. Let $a \geq 1$ and $b > 1$ be constants, let $f(n)$ be a function and let $T(n)$ be defined on the nonnegative integers by the recurrence

$$T(n) = aT(n/b) + f(n), \tag{13}$$

where we interpret n/b to mean either $\lfloor n/b \rfloor$ or $\lceil n/b \rceil$. Then $T(n)$ has the following asymptotic bounds:

1. If $f(n) \in \mathcal{O}\left(n^{\log_b(a)-\epsilon}\right)$ for some constant $\epsilon > 0$, then $T(n) = \Theta\left(n^{\log_b(a)}\right)$.
2. If $f(n) = \Theta\left(n^{\log_b(a)}\right)$, then $T(n) = \Theta\left(n^{\log_b(a)} \log n\right)$.
3. If $f(n) = \Omega\left(n^{\log_b a+\epsilon}\right)$ for some constant $\epsilon > 0$, and if $af(n/b) \leq cf(n)$ for some constant $c < 1$ and all sufficiently large n, then $T(n) = \Theta(f(n))$.

SUPPLEMENTARY NOTE 3 - CIRCUIT SIZE

Let us show that the size $S(n)$ of a $C^n(X)$ gate is $\tilde{\Theta}(n)$. Clearly, $S(n) \in \Omega(n)$. Let us show that $S(n) \in \mathcal{O}\left(n \log(n)^4\right)$. Let $n = 2^{k+2}$. Then,

$$S\left(2^{k+2}\right) \leq 4\left(2^{k/2+2} S\left(2^{k/2+2}\right) + 1\right). \tag{14}$$

Define $\tilde{S}(n) \equiv S(n)/n$. Then,

$$\tilde{S}\left(2^{k+2}\right) \leq 16\tilde{S}\left(2^{k/2+2}\right) + \mathcal{O}(1) \in \mathcal{O}\left(k^4\right). \tag{15}$$

As a consequence, $S(n) \in \mathcal{O}\left(n \log(n)^4\right)$ and $S(n) \in \tilde{\Theta}(n)$.

[1] T. H. Cormen, C. E. Leiserson, R. L. Rivest, and C. Stein, Introduction to Algorithms, Third Edition, 3rd ed. (The MIT Press, 2009).
